# Uterine giant cell carcinoma: a case report and review of the literature

**DOI:** 10.4274/tjod.galenos.2018.31855

**Published:** 2019-03-27

**Authors:** Hülya Ayık Aydın, Hasan Aykut Tuncer, Gülgün Erdoğan, Tayup Şimşek

**Affiliations:** 1 Akdeniz University Faculty of Medicine, Department of Obstetrics and Gynecology, Division of Gynecologic Oncological Surgery, Antalya, Turkey; 2 Akdeniz University Faculty of Medicine, Department of Pathology, Division of Gynecopathology, Antalya, Turkey

**Keywords:** Endometrial carcinoma, giant cell tumor, uterus

## Abstract

Endometrial carcinoma is the most common genital malignancy in women. Endometrioid type is the most common variant of endometrial carcinoma described in literature. Giant cell carcinoma is a rare, and infrequently reported variant of endometrial carcinoma. We present a 75-year-old patient admitted with vaginal bleeding. Transvaginal ultrasound revealed a 26x28 mm hypodense lesion without any adnexal pathology. The patient underwent total abdominal hysterectomy, bilateral salpingo-oophorectomy, and bilateral pelvic, and paraaortic lymph node dissection. The final histopathology report indicated a 3.8x2x9 cm giant cell carcinoma variant of endometrial carcinoma and one positive external iliac lymph node metastasis. Administration of adjuvant carboplatin and paclitaxel chemotherapy was given. As far as we know, this is the fifteenth case reported in the English literature.

## Introduction

Abnormal vaginal bleeding is the most common cause of referrals to gynecology outpatient clinics^(1)^. In women age 40-50 years, the endometrial cancer (EC) incidence was 13.6-24 in 100,000 women, and 87.3 in 100,000 women in the 70-74 years age group^(2)^. EC is the 4^th^ most common genital cancer in women, and endometrioid type accounts for 80% of all ECs^(3)^. Rare, and infrequently reported variants of EC include hepatoid carcinoma, glassy cell carcinoma, lymphoepithelioma-like carcinoma, adenocarcinoma with trophoblastic differentiation, and giant cell carcinoma (GCC)^(3)^. However, infrequent variants are under-reported in the English literature.

Nash and Stout^(4)^ described GCC in 1958 to define an aggressive cancer of the lung. GCC is a recently defined variant of EC. It is a unique and rarely described entity with only 14 cases reported in the literature to date^(5,6,7,8,9)^. Consequently, even though this tumor appears to have aggressive behavior in particular cases, the prognosis of GCC remains uncertain.

Herein, we aimed to present a rare case of uterine GCC in a 75-year-old female.

## Case Report

**Figure 1:**
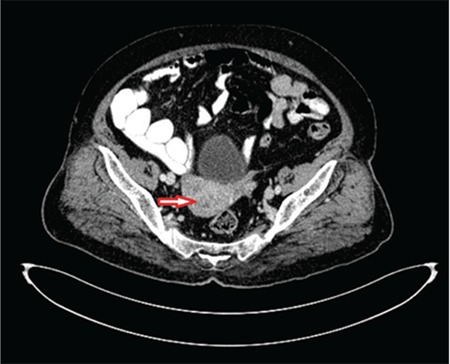
Abdominal computed tomography showing heterogeneous appearance in the endometrial cavity

A 75-year-old G5P5 patient who had been postmenopausal for 23 years was admitted with symptoms of vaginal bleeding. The patient additionally had type 2 DM and hypertension. A gynecologic examination revealed normal external genitalia, atrophic collum, intact adnexa, and free parametrium. Laboratory test results were as follows: CA125: 82 U/mL, CA19-9: 42 U/mL, and glycated hemoglobin (HbA1c): 11%. Transvaginal ultrasound revealed linear endometrium, minimal intracavitary fluid, and a 26x28 mm hypodense lesion extending to the serosa with no adnexal pathology. Abdominal computed tomography revealed no pathology in the liver, spleen, kidney, small and large bowels, and ovarian loge. Endometrial cavity had a heterogeneous appearance, and no intra- and retro- peritoneal pathologic lymph node was detected (Figure 1).

**Figure 2:**
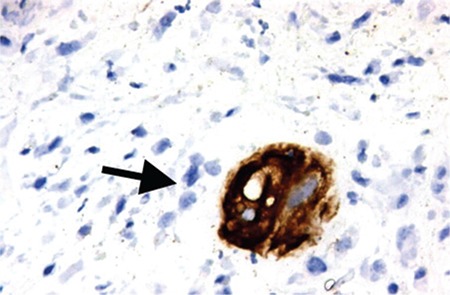
Immunohistochemistry PanCk positive staining of the tumor giant cells (x400)

**Figure 3:**
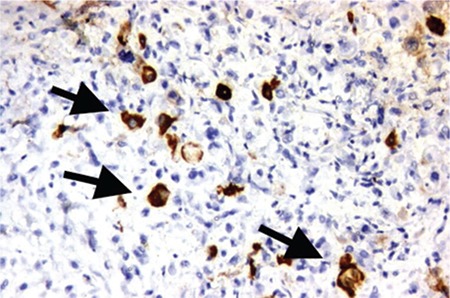
Immunohistochemistry PanCk positive staining of the tumor giant cells (x200)

**Figure 4:**
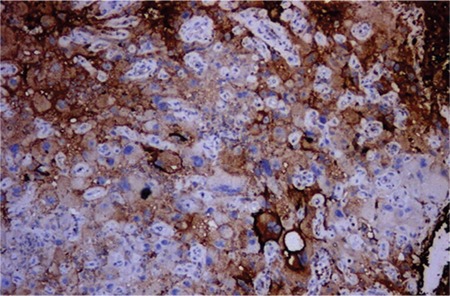
Immunohistochemistry vimentin positive staining of the tumor (x200)

Endometrial biopsy established the diagnosis of mixed EC [GCC (structural grade 3, and nuclear grade 3), and EC (structural grade 2, nuclear grade 2)]. Immunohistochemically, vimentin, and EMA produced widespread staining in the lesion (Figure 2). The histologic feature is bizarre multinucleated giant cells admixed with mononucleate tumor cells (Figures 3 and 4). Both tumors were stained with P53 focally, and ER dye stained areas of the EC. The tumor did not stain with P16, CEA, beta HCG and P63, desmin, MyoD1, CD10, caldesmon, and cyclinD1.

The results of cytokeratin staining were as follows: microscopic examination revealed large geographic tumor necrosis, multinuclear and mononuclear giant cells, and atypical mitosis. Therefore, endometrial neoplasms involving giant cells were considered and differential diagnosis included carcinoma, carcinosarcoma, leiomyosarcoma with osteoclast-like giant cells, undifferentiated sarcoma and choriocarcinoma with osteoclast-like giant cells. B-HCG was administered immunohistochemically and a negative reaction was observed. AE1/AE3 also showed a positive reaction in giant cells.

The patient underwent laparotomy, total abdominal hysterectomy, bilateral salpingo-oophorectomy, omental biopsy, and bilateral pelvic, and paraaortic lymph node dissection. The intraoperative frozen section result was reported as a tumor with a size of 3.8 cm, and more than half of the myometrium was invaded. Postoperative follow-up of the patient was uneventful, so she was discharged. The final histopathology report indicated a 3.8x2x9 cm GCC variant of EC and one positive external iliac lymph node metastasis. Cytology of intraabdominal specimens was unremarkable. Administration of adjuvant carboplatin and paclitaxel chemotherapy was planned upon the decision of the multidisciplinary council.

## Discussion

This is a unique case presenting a GCC of the endometrium. GCC is a rare and aggressive endometrial variant that was first described in 1991 by Jones et al.^(5)^. As far as we know, this is the 15^th^ case reported in the English literature.

Endometrial sampling should be performed on all women aged over 45 years who are suspected of having anovulatory uterine bleeding^(6)^. Postmenopausal bleeding is the most common symptom of EC, which is detected in 10-15% of cases^(7)^. Therefore, patients with postmenopausal bleeding should be evaluated in detail. The ages of all patients reported in the literature so far ranged from 43 to 85 years. Jones et al^(5)^. reported six patients with uterine GCC. All patients presented with vaginal bleeding. Occasional giant cells were positive for CK and EMA, whereas desmin and SMA were negative in all cases. Of the first six patients reported in the literature, four developed recurrence and three died in 3 years. Mulligan et al^(8)^. reported five patients, three of whom admitted with vaginal bleeding, one with anemia, and one with a pelvic mass. Of these five patients, one was disease-free after 14 years, three patients showed no symptoms or signs related to the disease during the 15 to 32-month follow-up period, and one patient had metastasis to the lung four years after diagnosis. Bhattacharyya et al. reported a 70-year-old postmenopausal patient with symptoms of vaginal bleeding^(9)^. Johannesen et al.^(10)^ reported a 70-year-old woman with postmenopausal bleeding. Sharma et al.^(11)^ presented a 60-year-old patient with vaginal bleeding. In the present case, a 75-year-old G5P5 postmenopausal patient admitted with symptoms of vaginal bleeding.

Endometrial carcinoma diagnosis should be verified by curettage and histopathologic examination of the tissue, as performed in the present case, also, when the final diag­nosis can only be achieved in the surgical specimen^(7)^. Precise classification is mandated because the histologic type complemented by staging is crucial in the selection of treatment of choice^(8,9)^. The present case was categorized as International Federation of Gynecologists and Obstetricians stage 3C1. Accurate classification should be accomplished through histopathological examination, which is the gold standard.

The surgical treatment of EC is panhysterectomy with or without pelvic and paraaortic lymphadenectomy depending upon the grade of the tumor. Hormone therapy in EC is a well-established treatment modality for primary, metastatic, and recurrent cases. However, the role of hormone therapy in this rare and aggressive subtype of EC remains unstudied. To date, there are no data on the receptor (ER and PR) status of this tumor, positivity of which in our study could have a therapeutic implication.

Bhattacharyya et al.^(9)^, Johannesen et al.^(10)^, and Sharma et al.^(11)^ all reported total hysterectomy and bilateral salpingo oophorectomy as the treatment of choice in their patients who were reported to have been followed up in the outpatient clinic with no complaints. The present patient underwent total abdominal hysterectomy, bilateral salpingo oophorectomy, and bilateral pelvic and para-aortic lymphadenectomy.

## Conclusion

In conclusion, GCC is a rare, and infrequently reported variant of EC diagnosed through histopathologic examinations of resected specimens. Awareness of this subtype of EC is essential to avoid misclassification of these cases due to a wide variety of differential diagnoses and poor prognosis.
